# Regenerative Medicine in Diabetes

**DOI:** 10.3390/biomedicines8120537

**Published:** 2020-11-25

**Authors:** Shoichiro Sumi

**Affiliations:** Institute for Frontier Life and Medical Sciences, Kyoto University, 53 Shogoin-Kawara-cho, Sakyo-ku, Kyoto 606-8507, Japan; sumi@infront.kyoto-u.ac.jp

Diabetes mellitus (DM) is caused by insufficient insulin function [1]. Since the purpose of regenerative medicine is to cure diseases or injuries by reconstructing lost forms and/or functions, the goal of regenerative medicine in relation to diabetes is, therefore, to cure DM by regaining insulin function in the body of DM patients. Loss of insulin function is usually caused by decreased insulin sensitivity and relatively insufficient insulin secretion in type 2 DM (T2D) or absolute secretory defect of insulin due to autoimmune destruction of insulin-secreting β-cells in type 1 DM (T1D). Therefore, as Dr. Banting stated almost a century ago in a Nobel lecture [2], exogenous insulin administration is not a cure for diabetes but a treatment for T1D patients. In that sense, transplantation of the pancreas organ or isolated islets can cure DM by re-establishing insulin action. However, current transplantation therapy for DM needs immunosuppression and human donors [3]. Regenerative medicine should achieve similar effects without the necessities of immunosuppression and human cadavers. Regenerative medicine may also prevent the autoimmunity and/or islet disfunction that develops DM. Recent advances in regenerative medicine for diabetes are briefly summarized in this very short review. 

Considering therapies for endocrine and metabolic diseases, the site where a therapeutic device is placed is not limited to the organ or tissue that causes the disease but anywhere in the body as long as the device can access general circulation. Therefore, islets are usually transplanted to the liver via portal vein, and pancreas is usually transplanted to the right lower abdomen with anastomoses to the iliac artery and vein.

Insulin-producing β-cells can be obtained by differentiating pluripotent and other stem cells [4,5,6]. Xenogeneic islets can be obtained from pigs [7], and even human islet cells may be made in pigs by blastocyst complementation technology, although the studies seem to stop at rodent models [8]. However, these cells need immunosuppression if they are transplanted without immune isolation, unless the cells originated from a patient’s own cells. Therefore, immune isolation technology is important to avoid immunosuppression. 

Since 1980, when the first successful microencapsulation of the islet was reported [9], many immune isolation methods such as chambers, blood perfusing methods, and encapsulation methods have been reported as bioartificial pancreases [10]. Among them, microencapsulation has been the most studied, and some bioartificial pancreases have been used for humans [11,12], but their efficacy has been erratic due to the tight packing of the transplanted beads and/or fibrous capsule formation around the beads due to foreign body reaction [12]. Effective control of foreign body reaction is really important for long-term function of a transplanted bioartificial pancreas. For example, our long-term (24 weeks) observation following intraperitoneal transplantation of macroencapsulated islets embedded in polyvinyl alcohol hydrogel in rats showed a gradual increase in blood sugar, and the retrieved device showed a thin but secure formation of fibrous membrane on the surface [13]. However, in the case of microencapsulation, it is not easy to control, since materials that should become hydrogels via a non-cytotoxic change of the environment (usually ions or temperature) are limited. In addition, microencapsulated islets cannot be fully retrievable. On the other hand, a macroencapsulation device can be fully retrievable and exchangeable, if necessary, and materials can be selected and/or biologically manipulated. 

A clinical trial of the macroencapsulated human islets using the βAir device that needs daily oxygen injection has been reported [14]. The efficacy of the transplantation was minute due to the fibrotic membrane formation around the device, although the device allowed islet survival. Therefore, we proposed a highly porous EVOH (ethylene vinyl-alcohol co-polymer) bag with high biocompatibility [15], in which immunoisolating hydrogel-embedding islets were packed. The intraperitoneal transplantation of rat islets embedded in chitosan-gel and packed in an EVOH bag successfully controlled mouse DM and the retrieved device showed minimal adhesion due to foreign body reaction [15]. Very recently, co-encapsulation of hepatocyte growth factor (HGF) with islets enabled primary (without pre-treatment) subcutaneous transplantation of a similar device [16]. However, in the long-term experiment, the retrieved device showed very little immunoisolating gel in the EVOH bag. Therefore, now, we are seeking a more durable hydrogel in vivo. Progress in immune isolation technologies will enable islet replacement therapy without immune suppression. 

A recent clinical study suggested that cell-based therapy can cure the autoimmunity that causes T1D [17], and β-cell function was maintained in patients with residual β-cell function but not in those without it. The researchers used Stem Cell Educator (SCE) therapy, which used human allogeneic cord blood-derived multipotent stem cells. In general, mesenchymal stem cells (MSCs) have immunomodulatory function as well as differentiation potential, and may be useful treating DM and hopefully curing T1D [18]. 

Regenerative medicine using living cells is being actively studied in the world and is creating brilliant results, especially for diseases that have been previously untreatable. This is also true in DM therapy, for which β-cell replacement without immunosuppression and control of the autoimmunity that causes T1D are getting closer to becoming a reality.

## References

[1] Petersmann A., Müller-Wieland D., Müller U.A., Landgraf R., Nauck M., Freckmann G., Heinemann L., Schleicher E. (2019). Classification and Diagnosis of Diabetes Mellitus. Exp. Clin. Endocrinol. Diabetes.

[2] Frederick G. Banting Nobel Lecture. https://www.nobelprize.org/prizes/medicine/1923/banting/lecture/.

[3] Niclauss N., Meier R., Bédat B., Berishvili E., Berney T. (2016). Beta-Cell Replacement: Pancreas and Islet Cell Transplantation. Endocr.

[4] Veres A., Faust A.L., Bushnell H.L., Engquist E.N., Kenty J.H., Harb G., Poh Y.C., Sintov E., Gürtler M., Pagliuca F.W. (2019). Charting cellular identity during human in vitro β-cell differentiation. Nature.

[5] Millman J.R., Xie C., Van Dervort A., Gürtler M., Pagliuca F.W., Melton D.A. (2016). Generation of stem cell-derived β-cells from patients with type 1 diabetes. Nat. Commun..

[6] Millman J.R., Pagliuca F.W. (2017). Autologous Pluripotent Stem Cell-Derived β-Like Cells for Diabetes Cellular Therapy. Diabetes.

[7] Dhanasekaran M., George J.J., Loganathan G., Narayanan S., Hughes M.G., Williams S.K., Balamurugan A.N. (2017). Pig islet xenotransplantation. Curr. Opin. Organ. Transplant..

[8] Kobayashi T., Yamaguchi T., Hamanaka S., Kato-Itoh M., Yamazaki Y., Ibata M., Sato H., Lee Y.S., Usui J.I., Knisely A.S. (2010). Generation of rat pancreas in mouse by interspecific blastocyst injection of pluripotent stem cells. Cell.

[9] Lim F., Sun A.M. (1980). Microencapsulated islets as bioartificial endocrine pancreas. Science.

[10] Sumi S. (2011). Regenerative medicine for insulin deficiency: Creation of pancreatic islets and bioartificial pancreas. J. Hepatobiliary Pancreat. Sci..

[11] Matsumoto S., Tan P., Baker J., Durbin K., Tomiya M., Azuma K., Doi M., Elliott R.B. (2014). Clinical porcine islet xenotransplantation under comprehensive regulation. Transplant. Proc..

[12] Tuch B.E., Keogh G.W., Williams L.J., Wu W., Foster J.L., Vaithilingam V., Philips R. (2009). Safety and viability of microencapsulated human islets transplanted into diabetic humans. Diabetes Care.

[13] Qi Z., Yamamoto C., Imori N., Kinukawa A., Yang K.C., Yanai G., Ikenoue E., Shen Y., Shirouzu Y., Hiura A. (2012). Immunoisolation effect of polyvinyl alcohol (PVA) macroencapsulated islets in type 1 diabetes therapy. Cell Transplant..

[14] Carlsson P.O., Espes D., Sedigh A., Rotem A., Zimerman B., Grinberg H., Goldman T., Barkai U., Avni Y., Westermark G.T. (2018). Transplantation of macroencapsulated human islets within the bioartificial pancreas betaAir to patients with type 1 diabetes mellitus. Am. J. Transplant..

[15] Yang K.C., Yanai G., Yang S.Y., Canning P., Satou Y., Kawagoe M., Sumi S. (2018). Low-adhesive ethylene vinyl alcohol-based packaging to xenogeneic islet encapsulation for type 1 diabetes treatment. Biotechnol. Bioeng..

[16] Yang S.Y., Yang K.C., Sumi S. (2020). Prevascularization-free Primary Subcutaneous Transplantation of Xenogeneic Islets Coencapsulated With Hepatocyte Growth Factor. Transplant. Direct..

[17] Delgado E., Perez-Basterrechea M., Suarez-Alvarez B., Zhou H., Revuelta E.M., Garcia-Gala J.M., Perez S., Alvarez-Viejo M., Menendez E., Lopez-Larrea C. (2015). Modulation of autoimmune T-cell memory by stem cell educator therapy: Phase 1/2 clinical trial. EBioMedicine.

[18] Wu H., Mahato R.I. (2014). Mesenchymal stem cell-based therapy for type 1 diabetes. Discov. Med..

